# Continuous Glucose Monitoring of Steroid-Induced Hyperglycemia in Patients With Dermatologic Diseases

**DOI:** 10.1177/19322968221147937

**Published:** 2023-01-05

**Authors:** Monika Kleinhans, Lea Jessica Albrecht, Sven Benson, Dagmar Fuhrer, Joachim Dissemond, Susanne Tan

**Affiliations:** 1 Department of Dermatology, Allergology and Venerology, University Hospital Essen, University of Duisburg-Essen, Essen, Germany; 2 Institute of Medical Psychology and Behavioral Immunobiology & Institute for Medical Education, Center for Translational Neuro- and Behavioral Sciences, Medical Faculty, University Hospital Essen, University of Duisburg-Essen, Essen, Germany; 3 Department of Endocrinology, Diabetes and Metabolism, University Hospital Essen, University of Duisburg-Essen, Essen, Germany

**Keywords:** continuous glucose monitoring, diabetes mellitus, glucocorticoid, steroid-induced diabetes, steroid-induced hyperglycemia

## Abstract

**Background and Objectives:**

Systemic administration of glucocorticoids is a mainstay therapy for various inflammatory diseases and may lead to hyperglycemia, which carries the risk of worsening preexisting diabetes and triggering steroid-induced diabetes. Therefore, we aimed to identify patients at risk and to quantify severity of steroid-induced hyperglycemia (SIH) by continuous glucose monitoring (CGM) in hospitalized patients needing systemic glucocorticoid treatment.

**Patients and Methods:**

This prospective study included 51 steroid-naive, dermatological patients requiring systemic high-dose glucocorticoid treatment at the Department of Dermatology of the University Hospital Essen. After careful diabetes-specific assessment at admission, glucose monitoring was performed using a CGM system and glucose profile was analyzed in patients with and without SIH.

**Results:**

SIH occurred in 47.1% of all treated patients, and a relevant part of patients with initial normoglycemia developed SIH (2/10 patients). Doubling of SIH incidence was observed with each severity grade of dysglycemia (4/10 in prediabetes; 9/10 in diabetes). Patients with SIH spend nearly 6 hours daily above targeted glucose range, and severe hyperglycemia was observed for 1.2 hours/day.

**Conclusions:**

Our study underlines the need for dedicated glucose monitoring in dermatologic patients on systemic glucocorticoid therapy by demonstrating its impact on glucose metabolism.

## Introduction

Systemic administration of glucocorticoids presents a mainstay treatment in various inflammatory processes including various dermatological diseases.^1^ However, glucocorticoids may lead to hyperglycemia by enhancing insulin resistance in liver, muscle, and adipose tissue and by increasing hepatic gluconeogenesis.^2^ In general, blood glucose levels peak four to six hours after, eg, prednisolone administration and may persist for up to 12-24 hours.^3^ This effect can both worsen preexisting diabetes and trigger steroid-induced diabetes.^4^ The prevalence of the use of systemic glucocorticoids varies. For example, 1.2% of adults in the United States received long-term systemic glucocorticoid therapy in 2013,^5^ and 3% of the Danish population had a prescription for systemic glucocorticoids at least once a year during 1999 to 2015.^6^ In hospitalized patients, the prevalence of glucocorticoid therapy patients is assumed to be 10%.^2^

The prevalence of steroid-induced diabetes is not precisely known; however, literature indicates an incidence of approximately 12%.^7^ Glucocorticoids are a common cause of new-onset hyperglycemia in hospitalized patients, and new-onset hyperglycemia is associated with an increased risk of morbidity and mortality compared with chronic hyperglycemia.^8^ The HbA1c value as marker of chronic hyperglycemia reflects the mean blood glucose of the past 8 to 12 weeks. HbA1c is not only used to assess quality of glycemic control in patients with known diabetes but also as a diagnostic tool. For example, mild elevation of HbA1c of (5.7%-6.4%) reflects prediabetes, while HbA1c values of 6.5% and higher define overt diabetes mellitus. Thus, HbA1c is helpful to assess patient’s baseline diabetes status before the exposure to glucocorticoids. However, in case of steroid triggered new-onset diabetes, HbA1c is not a reliable diagnostic parameter.^9^ Hence, blood glucose monitoring is recommended before meals or every 4 to 6 hours.^10^ There is no uniform consensus in the current guidelines on monitoring patients during systemic glucocorticoid therapy. Although the German working group of scientific medical societies guideline (Arbeitsgemeinschaft der Wissenschaftlichen Medizinischen Fachgesellschaften) states that the indication of insulin should be reviewed during systemic glucocorticoid administration, it does not provide any recommendations on the frequency of blood glucose measurement.^11^

Identification of patients at risk for steroid-induced hyperglycemia (SIH), as well as the high rate of patients with known and unknown prediabetes and diabetes in the general population, presents daily challenges in clinical practice. More importantly, it must be considered that patients receiving steroids in the morning have disproportionate hyperglycemia during the day but normal blood glucose levels overnight and in the morning at the time of steroid application. In addition to the need for glucose monitoring, antidiabetic treatment must also be adapted to anticipated changes in glucocorticoid doses. Thus, we performed a diabetes-specific assessment of patients needing in-hospital high-dose glucocorticoid treatment by systematic diabetes screening and continuous glucose monitoring to identify patients at risk for and to quantify severity of SIH.

## Materials and Methods

### Patient Cohort

This prospective study was performed between April 2021 and November 2021 at the Department of Dermatology of the University Hospital Essen and included 51 steroid-naive in-patients with dermatologic disease requiring systemic high-dose glucocorticoid treatment. Treatment indications included, eg, bullous pemphigoid, cutaneous leukocytoclastic angiitis, and drug-related exanthema but also rare inflammatory dermatological diseases. Patients on high-dose steroid treatment both with and without tapering were included. High-dose glucocorticoid treatment was initiated with prednisolone 0.75 to 2.0 mg/kg bodyweight (bw) per day intravenously (IV). Patients who had received any kind of glucocorticoid therapy previously or who were on glucocorticoid therapy that had been started before admission were excluded.

### Data Collection

Anthropometric data such as age, height, weight, body mass index (BMI), as well as information on insulin or other antidiabetic medication prior to admission were extracted from the electronic health records. Blood examination including diabetes-specific parameters such as HbA1c, random glucose, C-peptide, and insulin were obtained for each patient at admission. Glucose monitoring was performed by using a continuous glucose monitoring system (CGM).

### Diabetes-Specific Assessement

To evaluate patient’s glycemic status, all patients were screened systematically for dysglycemia by measurement of HbA1c and random blood glucose as well as by personal interview and electronic search for diabetes-specific International Statistical Classification of Diseases and Related Health Problems (ICD) codes documented in previous in-hospital stays (prediabetes: R73.0, diabetes: E10-14.0, e.g. E11 = type 2 diabetes). Diabetes was defined by HbA1c ≥ 6.5% (47.5 mmol/mol ), random plasma glucose ≥ 200 mg/dL (11.1 mmol/L), or known history of diabetes. Prediabetes was defined by HbA1c 5.7% to 6.4% (38.8 - 46.5 mmol/mol ) and/or known history of prediabetes. Patients with random plasma glucose < 200 mg/dL (11.1 mmol/L) and HbA1c < 5.7% (38.8 mmol/mol) and absence of known history of diabetes and prediabetes were classified as normoglycemic. Method of HbA1c measurement fulfilled the standards of “National Glycohemoglobin Standardization Program (NGSP; DCCT-aligned)”. To detect hyperglycemia, glucose monitoring under steroid exposition was performed by a CGM . The CGM sensor (FreeStyle Libre 2/3 [FSL], Abbott) was implanted subcutaneously prior to initiation of systemic glucocorticoid therapy. It measured tissue glucose continuously (once in a minute) delivering 1440 glucose values per day for up to 14 days, including hyperglycemia and hypoglycemia alarm as well as arrows predicting glucose trend. Glucose monitoring was started at admission and continued until discharge. Data were transmitted via near field contact (FSL2) resp. Bluetooth (FSL 3) to an application on a smartphone (FreeStyle LibreLink, Abbott) and shared in real time with nursing staff from application to application (FreeStyle LibreLinkUp, Abbott) and with the diabetes team using a cloud-based glucose management system (Libre View, Abbott). CGM data were available as absolute values of measured glucose during the in-hospital stay and as ambulatory glucose profile. SIH was defined as presence of recurrent glucose values ≥ 200 mg/dL (11.1 mmol/L) under exposition of glucocorticoid treatment during the in-hospital stay. No or only one glucose reading ≥ 200 mg/dL (11.1 mmol/L) were regarded as absence of SIH. To quantify the influence of glucocorticoid exposition on glucose metabolism in more detail, the following parameters were used to phenotype included patients individually with regard to glucose control: time in range (TIR, glucose 70-180 mg/dL, 3.9-10 mmol/L, target 70%), time above range (TAR-high, glucose 180-250 mg/dL, 10-13.9 mmol/L, target: <5%, TAR-very high, glucose > 250 mg/dL, > 13.9 mmol/L, target < 5%), time below range (TBR, glucose < 70 mg/dL, < 3.9 mmol/L, target < 5%), glucose variability (GV, target ≤ 36%), mean glucose (MG, target < 154 mg/dL, < 8.5 mmol/L), and glucose management index (GMI, target < 7.0%).

### Statistical Analysis

For risk estimation and identification of patients at high risk for SIH, screening results and glucose profile details obtained by CGM were analyzed in patients with SIH and without steroid-induced hyperglycemia (NoSIH). All data are presented as mean ± standard deviation unless otherwise stated. Group comparisons were analyzed using the non-parametric Mann-Whitney *U* test for continuous data. Frequency differences for dichotomous variables were calculated using chi-square test. The alpha error was set at α = 0.05. Analyses were performed using the SPSS software (IBM Corporation, Armonk, NY, USA, version 27).

### Ethics

The study was approved by the ethics committee of the University of Duisburg-Essen and was performed in accordance with the Declaration of Helsinki (approval number 20-9333-BO).

## Results

### Patient Cohort Characteristics

Patients with incomplete dysglycemia screening and/or glucose monitoring other than CGM were excluded from analysis. A total of 51 of 56 patients (30/51 female, mean age 67 ± 18.5 years, mean BMI 29 ± 7.5 kg/m²) were included. Common diagnoses on admission were bullous pemphigoid (N = 14, 27.5%), drug-related exanthema (N = 10, 19.6%), leukocytoclastic vasculitis (N = 6, 11.8%), atopic eczema (N = 4, 7.8%), erythema nodosum (N = 3, 5.8%), lichen planus (N = 2, 3.9%), and pyoderma gangrenosum (N = 2, 3.9%). Rarer admission diagnoses (N = 1, 2% each) were cheiropodopompholyx, Chilblain’s lupus, lupus erythematosus tumidus, Sweet’s syndrome, pemphigus foliaceus, pityriasis rubra pilaris, prurigo nodularis, psoriasis pustulosa generalisata, and erythema exsudativum multiforme. The most frequently administered glucocorticoid dose was prednisolone 1 mg/kg bw (N = 36, 70.6%). Other doses administered were 0.75 mg/kg bw (N = 12, 23.5%), 0.5 mg/kg bw (N = 1, 2%), 1.25 mg/kg bw (N = 1, 2%), and 2 mg/kg bw (N = 1, 2%).

### Diabetes-Specific Phenotype

Prior to glucocorticoid exposition, mean HbA1c was 6.1 ± 1.3% (range: 4.6%-11%) resp. 43 (range: 27 – 97 mmol/mol). Based on HbA1c, initial prevalence of no diabetes (NoD), prediabetes (PreD), and diabetes (D) was 35.3% (N=18), 37.3% (N=19), and 27.5% (N=14), respectively. At baseline, random glucose was 133 ± 82 mg/dL (7.4 ± 4.6 mmol/L). During the treatment period, patients’ glucose levels were monitored for 7 ± 2 days (range: 2-14). Glucose levels ranged from 72 to 506 mg/dL (4-28.1 mmol/L ). Mean TIR, TAR, and TBR were 82.9 ± 11.7%, 8.8 ± 11.7%, and 2.6 ± 6.9%. MG was 123 ± 31 mg/dL (6.3 ± 1.7 mmol/L) (range: 80-213 mg/dL, 4.4 ± 11.8 mmol/L). GMI could only be calculated for 19 of 51 (37.3%) patients, since not all patients were monitored for a sufficiently long period to allow algorithm-based GMI calculation on basis of MG. In those patients with available data, GMI was 6.4 ± 0.7% (range: 5.6-8.0).

### Group Comparison of Patients With and Without Steroid-Induced Hyperglycemia

**Figure 1.**
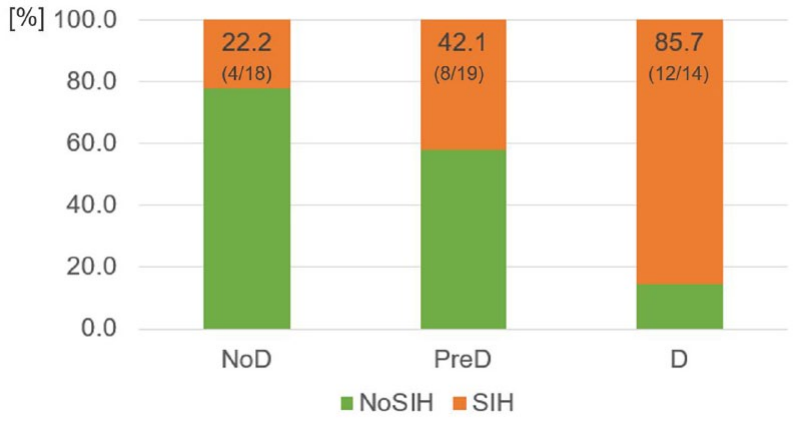
Incidence of SIH according to glycemic status at admission (χ² = 13.04; p=0.001). Data are presented as percentage affected (absolute numbers). NoD: no diabetes, PreD: prediabetes, D: Diabetes, NoSIH: no steroid-induced hyperglycemia, SIH: steroid-induced hyperglycemia.

**Figure 2.**
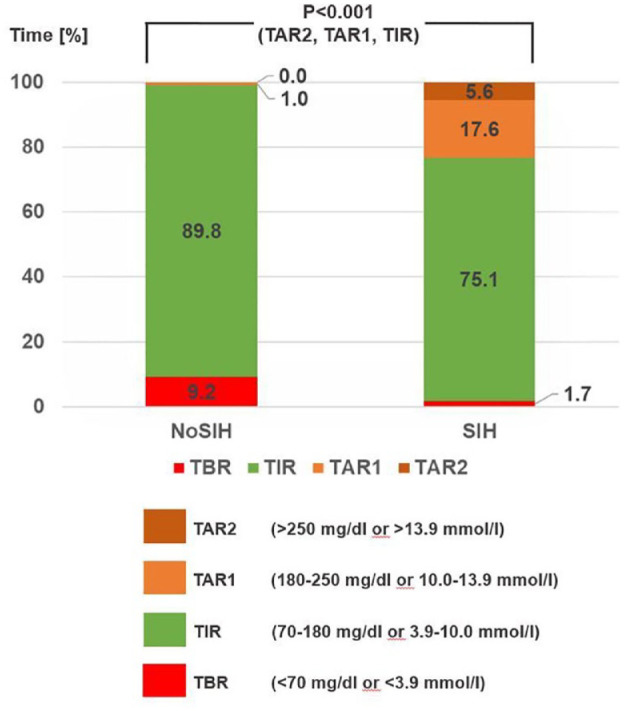
Glucose profile of patients without (NoSIH) and with steroid-induced hyperglycemia (SIH) derived by a continuous glucose monitoring system. Data are presented as percentage of daily time spent below range (TBR), in range (TIR), above range with high glucose values (TAR1) and above range with very high glucose values (TAR2). *P* values < .05 are regarded as significant.

Data on group analysis are presented in Table 1. In 24 of 51 patients (47.1%), SIH was observed, while 27 of 51 patients (52.9%) remained normoglycemic despite systemic exposition to glucocorticoids. The patient groups with and without SIH did not differ in age (*P* = .22), gender (*P* = .62), or BMI (*P* = .09). Furthermore, steroid dose applied and distribution of those with high-dose steroid therapy with and without tapering were comparable in both groups. Of note, incidence of SIH increased significantly with degree of dysglycemia status at admission (χ² = 13.04; *P* = .001) (Figure 1). SIH occurred in one of five patients with NoD (22.2%), in almost every second patient with PreD (42.1%) and in most patients with D (85.7%). All patients with D treated with insulin prior to admission developed SIH (100.0%) (χ² = 9.13, *P* = .003). Patients with NoSIH spent most of the day in target range. Patients with SIH spent at mean 4.2 hours daily in hyperglycemia with glucose values between 180 and 250 mg/dL (10 - 13.9 mmol/L) (*P* < .001) and at mean 1.3 hours per day in hyperglycemia with glucose values > 250 mg/dL (> 13.9 mmol/L) (Figure 2). Mean glucose (Z = 5.49, *P* < .001) and consequently GMI (ZU = 2.67, *P* = .008) but also GV (Z = 3.85, *P* < .001) were significantly higher in patients with SIH than in those with NoSIH.

**Table 1.** Patient Cohort Characteristics and Statistical Data.

	Patients without steroid-induced hyperglycemia (NoSIH) N = 27		Patients with steroid-induced hyperglycemia (SIH) N = 24		Test statistics*	*P*
Age, years	62.9 ± 19.9		70.7 ± 16.3		Z = 1.24	.22
Female, % [N]	50	[15]	50	[15]	χ² = 0.25	.62
Male, % [N]	57.1	[12]	42.9	[9]		
BMI (kg/m²)	27.5 ± 8.1		29.9 ± 6.8		Z = 1.70	.09
Days of CGM	6.8 ± 2.2		6.5 ± 2.5		Z = 0.88	.38
High-dose steroid treatment without tapering, % [N]	77.8	[7]	22.2	[2]	χ² = 2.71	.10
High-dose steroid treatment with tapering, % [N]	47.6	[20]	52.4	[15]		
Glucocorticoid dose, mg/kg/day	0.95 ± 0.21		0.96 ± 0.20		Z = 1.1	.26
Baseline HbA1c, %, mmol/mol	5.5 ± 0.4	36.61 ± −19.13	6.8 ± 1.6	50.82 ± −6.01	Z = 3.75	<.001
Baseline random glucose mg/dL, mmol/L	145.36 ± 107.09	8.08 ± 6.0	118.08 ± 30.97	6.6 ± 1.7	Z=0.03	.97
No Diabetes % [N]	77.8	[14]	22.2	[4]	χ² = 13,04	.001
Prediabetes % [N]	57.9	[11]	42.1	[8]		
Diabetes % [N]	14.3	[2]	85.7	[12]		
preadmission insulin use, % [N]	0.0	[0]	100.0	[7]	χ² = 9.13	.003
no preadmission insulin use, % [N]	61.4	[27]	38.6	[17]		
TAR-very high [%] (target: <5%)	0 ± 0		5.6 ± 9.3		Z = 4.1	<.001
TAR-high [%] (target: <20%)	1.0 ± 2.0		17.6 ± 11.9		Z = 6.09	<.001
TIR [%] (target: > 75%)	89.8 ± 10.6		75.1 ± 16.8		Z = 3.57	<.001
GV [%]	23.6 ± 5.3		30.7 ± 6.1		Z = 3.85	<.001
Mean glucose, mg/dL, mmol/L	102.6 ± 13.4	5.7 ± 0.7	145.6 ± 29.7	8.1 ± 1.6	Z = 5.49	<.001
Random glucose, mg/dL, mmol/L	101.8 ± 18.0	5.6 ± 1.0	167.4 ± 110.2	9.3 ± 6.1	Z = 3.28	.001
GMI,%	6.81 ± 0.21		6.01 ± 0.21		Z = 2.67	.008

Data are presented as mean ± standard deviation or percentage affected (absolute numbers). *Reported are results from U-test/Chi²-test as appropriate.

Abbreviations: SIH, steroid-induced hyperglycemia; BMI, body mass index; CGM, continuous glucose monitoring system; TAR, time above range; TIR, time above range; TBR, time below range; GV, glucose variability; GMI, glucose management indicator.

## Discussion

Although glucocorticoids significantly improve the outcome of many diseases due to their potent immunomodulatory and anti-inflammatory effects, undesirable side effects such as steroid-induced hyperglycemia may occur. We present the first study using a systematic diabetes screening and continuous glucose profiles to describe glycemic effects of high-dose steroid therapy in patients with inflammatory dermatologic disease. It could be demonstrated that SIH is a major health issue occurring in almost half of the patients under high-dosed steroid treatment and exposing this patient group to a marked TAR Level 1 and 2. Furthermore, SIH was characterized by high glycemic variability and elevation of GMI. In addition, this study pointed out the usefulness of a dysglycemia screening at admission to identify those patients at highest risk for SIH.

According to current literature, it can be assumed that in steroid-treated patients, 86% will experience at least one episode of hyperglycemia.^12^ Prevalence of SIH resp. steroid-induced diabetes is reported to be 39-78% and 40% in patients receiving steroids due to hematologic, rheumatologic or nephrologic indication. ^12-14^

To date, there are few data on the occurrence of steroid-induced diabetes in the context of dermatologic diseases, and studies are limited to the use of intermittent single glucose measurements. In a study including 30 dermatological patients, one third of patients with daily-administered steroids developed diabetes, while those on weekly medication never did.^15^ Our results show that a relevant part of patients with initial normoglycemia developed SIH (2 of 10 patients) and that doubling of SIH incidence occurred with each severity grade of dysglycemia (4 of 10 in prediabetes and 9 of 10 in diabetes). Assuming an incidence of 20% as relevant, glucose monitoring seems to be helpful in all patients, but dysglycemia screening at admission helps to identify those patients at even higher and highest risk. Of note, all patients with PreD and 20% of affected D patients in Germany are unaware of their dysglycemia status.^7^ Thus, without screening, these patients are more likely to remain undiagnosed and thus potentially undertreated.

In contrary to previous published data,^16^ in our cohort age and BMI were not associated with glucose derangement. This could be explained by methodological differences. As glucose was measured continuously, classification of patients as having NoSIH or SIH was made in our study based on substantial more glucose data in comparison to other studies using capillary glucose values. Thus, it can be assumed that the SIH group of this study included patients with milder forms of hyperglycemia, which could have been overlooked by an intermittent measuring approach. Patients with mild hyperglycemia are more often younger and less obese than those with overt diabetes. However, it cannot be excluded that missing significance could be explained by the relatively low number of patients included.

The heterogeneity of indications leading to steroid treatment, age of patients included, treatment duration, regimen, and dose applied may limit the comparability of our results to previous studies. However, our patient population reflects the real-world cohort of hospitalized patients needing high-dose steroid treatment for inflammatory dermatological diseases. Furthermore, patients who already had received steroid treatment prior to our study did not enter this study; hence, impact of prior existing steroid-induced influence on glucose metabolism could be excluded. As the observation period was limited to the in-hospital stay, SIH developed after discharge was not captured in our study, potentially leading to false low incidence rate of SIH. Diabetes, comorbidities, old age, and prolonged steroid use have previously been identified as relevant risk factors.^12^ Studies with CGM in the outpatient setting may elucidate the impact of steroids on SIH incidence in more detail.

In clinical practice, glucose monitoring in patients with SIH is performed with up to four capillary glucose values (before each meal and before bed). This limited number of information may not be sufficient to estimate the degree of hyperglycemia induced by steroid exposition. By continuous glucose monitoring SIH specific characteristics could be captured. It could be shown that patient with SIH spend nearly 6 hours daily above targeted glucose range with even 1.2 hours with more severe hyperglycemia. Not only reduction of time in normoglycemia but also a twofold higher variability of glucose values than in those with NoSIH was observed. A high variability is described as a prognostically unfavorable factor.^17^ Furthermore, occurrence of SIH was significantly associated with higher GMI. Thus, GMI considering glucose values of the past 14 days may be advantageous as marker to assess acute glucose derangement induced by steroid exposition in comparison to HbA1c which reflects glucose control of the past 6-12 weeks. In summary, our study describes in more detail the influence of high-dose steroids on glucose metabolism than usual capillary glucose values would have allowed. Additional advantages of the sensors are the lack of need for recurrent invasive fingerpricking and the quick and easy handling of CGM reducing disease management burden for the patients and workload for the nursing staff. Furthermore, CGM based hyper- and hypoglycemia alarm allows real-time intervention. However, the costs for consumables like the CGM sensor and the investment in smart devices cannot be ignored. Treating inpatient hyperglycemia by a sophisticated, efficient, and safe in-hospital diabetes management however may reduce in-hospital complications as well as diabetes diagnosis–associated financial burden. In addition, a diabetic metabolic situation leads to inflammation, induces skin lesions, and may worsen preexisting skin diseases.^18-20^ Fluctuations in blood glucose levels result in activation of the coagulation cascade, increased production of proinflammatory cytokines, induction of oxidative stress, elevation of LDL cholesterol, and endothelial dysfunction.^12^ Diabetes-specific intervention may reduce risk for adverse outcomes like nosocomial infection, length of stay, as well as in-hospital costs.^21-23^ In the outpatient setting, use of CGM has been shown to be associated with improvement of glucose control, increase in treatment satisfaction, and decrease in diabetes-related distress.^24,25^ Such data are not available for the hospital setting; however, one can assume that these findings may apply similarly to the in-hospital setting.

Further studies are warranted to examine the impact of SIH and SIH-management on dermatologic disease outcome in the short term and on metabolic complications in the long term, an aspect we have not addressed in our study.

## Conclusion

This clinical study reveals a high incidence of SIH under high-dose steroid therapy in dermatologic patients with no previous diabetes and usefulness of diabetes screening prior to steroid therapy for SIH risk estimation. This study underlines the need for glucose monitoring in patients on systemic glucocorticoid therapy. By using CGM, this study could precisely assess the impact of steroid application on glucose metabolism demonstrating a hitherto underestimated relevant period of hyperglycemia in affected patients. Furthermore, this study suggests CGM as a new tool to improve and simplify care of in-hospital patients at risk for hyperglycemia.
